# Metabolic signatures and potential biomarkers for the diagnosis and treatment of colon cancer cachexia: NMR metabolomic analysis of serum from cancer cachexia mice

**DOI:** 10.3724/abbs.2023151

**Published:** 2023-09-14

**Authors:** Xu Qiu, Ruohan Lu, Qiqing He, Shu Chen, Caihua Huang, Donghai Lin

**Affiliations:** 1 Key Laboratory for Chemical Biology of Fujian Province MOE Key Laboratory of Spectrochemical Analysis and Instrumentation College of Chemistry and Chemical Engineering Xiamen University Xiamen 361005 China; 2 Research and Communication Center of Exercise and Health Xiamen University of Technology Xiamen 361005 China

**Keywords:** cancer cachexia, serum, NMR-based metabolomics, metabolic profile, biomarker

## Abstract

Cancer cachexia (CAC) is a debilitating condition that often arises from noncachexia cancer (NCAC), with distinct metabolic characteristics and medical treatments. However, the metabolic changes and underlying molecular mechanisms during cachexia progression remain poorly understood. Understanding the progression of CAC is crucial for developing diagnostic approaches to distinguish between CAC and NCAC stages, facilitating appropriate treatment for cancer patients. In this study, we establish a mouse model of colon CAC and categorize the mice into three groups: CAC, NCAC and normal control (NOR). By performing nuclear magnetic resonance (NMR)-based metabolomic profiling on mouse sera, we elucidate the metabolic properties of these groups. Our findings unveil significant differences in the metabolic profiles among the CAC, NCAC and NOR groups, highlighting significant impairments in energy metabolism and amino acid metabolism during cachexia progression. Additionally, we observe the elevated serum levels of lysine and acetate during the transition from the NCAC to CAC stages. Using multivariate ROC analysis, we identify lysine and acetate as potential biomarkers for distinguishing between CAC and NCAC stages. These biomarkers hold promise for the diagnosis of CAC from noncachexia cancer. Our study provides novel insights into the metabolic mechanisms underlying cachexia progression and offers valuable avenues for the diagnosis and treatment of CAC in clinical settings.

## Introduction

Cancer cachexia (CAC) is a complex and multifactorial metabolic syndrome that involves numerous organs and metabolic changes. It is one of the leading causes of mortality in cancer patients, accounting for approximately 10% to 22% of all cancer deaths [1]. Patients suffering from cancer cachexia present with metabolic abnormalities, involuntary pathological body weight loss, fatigue, anemia, and loss of fat and skeletal muscle mass [ 1, 2]. Currently, the clinical classification of cancer cachexia development comprises three stages: the precachexia (noncachexia) stage, characterized by mild loss of appetite without significant body weight loss; the cachexia stage, characterized by significant body weight loss, along with skeletal muscle atrophy, multiple organ discomfort, loss of appetite, and other adverse signs; and the cachexia refractory stage, defined by physical condition deterioration, multiple organ failure, need for anticancer treatment, and other factors [ 3, 4] .

The development of CAC leads to rapid deterioration of patients’ physical condition, severely impacting their quality of life, reducing their tolerance to tumor treatment, and negatively affecting disease prognosis. Therefore, achieving accurate diagnosis and treatment of CAC at different stages is of utmost importance. There is an urgent need to identify metabolic changes occurring in the body during cachexia progression and explore potential biomarkers to assess the stage of cachexia and guide precise treatment [ 5, 6]. However, the current clinical diagnosis of cachexia mainly relies on body weight loss as the evaluation standard, which can result in inaccurate assessment of the degree of cachexia development [ 4, 7, 8].

The circulatory system is an all-encompassing reservoir for metabolites originating from various organs throughout the body. The intricate interplay of enzymatic reactions, physical factors, pathological conditions, and environmental influences collectively exerts a significant impact on metabolite levels in the sera, thereby influencing the overall metabolic profile. Comprehensively examining these metabolic changes can provide a deeper understanding of the underlying mechanisms and pathways involved in disease progression. This knowledge can pave the way for novel diagnostic approaches and therapeutic interventions aimed at targeting specific metabolic alterations, ultimately leading to improved patient care and treatment outcomes.

As a powerful tool for analyzing metabolic changes in biological fluids, metabolomics can provide theoretical and technical support for disease diagnosis and treatment. Among several techniques extensively used in metabolomics, nuclear magnetic resonance (NMR) spectroscopy has unique technical advantages, such as high reproducibility, objectivity, low sample requirements, and convenience for quantitative detection of metabolite concentrations [9]. Consequently, NMR-based metabolomic analysis has become a popular method for clarifying metabolic characteristics to mechanistically understand disease progression and identifying potential biomarkers to aid in disease diagnosis and treatment.

Numerous studies have explored potential biomarkers for cachexia using blood samples collected from patients or animal models. For instance, glucose has been identified as a biomarker for cancer development in various types of cancer [ 10, 11]. Myo-inositol has been proposed as a potential biomarker for the diagnosis of renal carcinoma [12], and glutamate and aspartate have been suggested as potential biomarkers for the diagnosis of esophageal squamous cell carcinoma [13]. These biomarkers may be useful for predicting the stage of cachexia and guiding precise treatment, providing clinicians with important information for the effective management of cachexia in cancer patients. However, further research is needed to validate these biomarkers and investigate their potential utility in clinical settings.

Despite the progress made in identifying cachexia biomarkers using blood samples, there are still limitations to the research. First, most studies have only categorized subjects into normal and cachexia groups, with a focus on screening biomarkers for disease diagnosis. However, cachexia involves dynamic metabolic changes during its progression. Previous studies on the same cancer type have yielded conflicting results. For example, Shin *et al*. [14] found a close association between early blood glucose elevation and the incidence of colorectal cancer, while Nishiumi *et al*. [ 15 , 16] observed significantly lower blood glucose level in patients with colorectal cancer compared to normal controls. A general definition and grouping fail to accurately determine the stage of cachexia and subsequently hinder precise treatment. Second, patients with different types of cancers exhibit diverse metabolic characteristics of cachexia, and studies that fail to differentiate between cancer types could result in confounded outcomes. For example, Yang *et al*. [17] investigated changes in serum metabolite content at different stages of cachexia, but the inclusion of patients with various cancer types, such as lung, liver, and stomach cancer, in the same group could mask characteristic changes specific to certain cancer types, potentially affecting the screening outcomes of biomarkers.

In this study, we established a mouse model of colon CAC through subcutaneous tumor implantation and performed NMR-based metabolomic analysis to investigate the metabolic characteristics of sera from three groups of mice, including CAC, noncachexia (NCAC) and normal controls (NOR) groups. Furthermore, we conducted multivariate ROC analysis to identify potential biomarkers in mouse sera for accurately distinguishing between CAC and NCAC stages during the progression of colon CAC. Our study offers novel insights into the metabolic mechanisms underlying cachexia progression and presents a promising tool for the diagnosis and treatment of CAC.

## Materials and Methods

### Cell culture

The CT26 cells were procured from the National Biomedical Cell Resource Bank (BMCR; Beijing, China). These cells were cultured in DMEM supplemented with 100 units/mL penicillin, 100 μg/mL streptomycin, and 10% fetal bovine serum (Hyclone, Logan, USA) under standard conditions. These conditions entailed incubation in a constant temperature incubator with 5% CO _2_ at 37°C. To maintain cell viability, the cells were subject to digestion with 0.25% trypsin-EDTA (Hyclone) within one hour of harvest. Thereafter, a cell suspension was prepared by diluting the cells in PBS buffer to attain the desired concentration. The cell concentration was determined by cell count.

### Animal experiments

The experimental animal protocol was approved by the Ethics Review Committee of Xiamen University (XMULAC20200150). All applicable institutional and governmental regulations concerning the ethical use of animals were followed.

To induce colon CAC in BALB/c mice, CT26 cells (1.0×10 ^6^/100 μL) were subcutaneously injected on day 0 ( Supplementary Figure S1) [18]. In the preliminary experiment conducted on tumor-bearing mice, we studied the time course of the onset and development of colon CAC in detail and determined the time points at which the mice entered NCAC and CAC to be 21 and 28 days, respectively. Therefore, NCAC ( *n*=12) mice were euthanized on day 21, and CAC mice ( *n*=12) were euthanized on day 28 in this study.

The daily food intake and body weights of the mice were monitored throughout the animal experiment. Tumor volumes were measured every three days using the following formula: tumor volume (mm ^3^)=0.52×length×width ^2^, with length and width measurements taken using a Vernier calliper. After euthanasia, both tumors and gastrocnemius muscles of the tumor mice were excised, weighed, and rapidly frozen in liquid nitrogen for subsequent analysis. Blood samples were collected in coagulation tubes, and serum was obtained by centrifugation (1000 *g*, 10 min at 4°C) within 2 h of collection.

In contrast, the normal control mice (NOR; *n*=11) were injected with an equal volume of PBS on day 0 ( Supplementary Figure S1) and did not exhibit any of the symptoms observed in the CAC and NCAC groups. All NOR mice were euthanized on day 28, and serum samples were collected for subsequent experiments.

By monitoring the food intake and body weights of the mice, we were able to determine the extent of cachexia-induced changes in the mice. This enabled us to investigate the impact of cachexia on the metabolism of the mice, as well as to identify potential biomarkers for the diagnosis and treatment of CAC.

### Inflammatory cytokine detection

Serum samples were assayed for the concentrations of inflammatory factors using an ELISA kit (SenBeiJia Biological Technology Co., Ltd., Nanjing, China). The optical density (OD) of the sample was measured at 450 nm using a microplate reader (PerkinElmer EnSpire, Shanghai, China), and the concentrations of inflammatory factors were calculated from the standard curve.

### Total antioxidant capacity test

The total antioxidant capacity of the gastrocnemius was determined using the ABST kit (Beyotime Biotechnology, Shanghai, China). ABST was used as the chromogenic agent, and the absorbance of ABST+ was measured at 734 nm or 405 nm using a microplate reader.

### NMR sample preparation

NMR-based metabolomic analysis was conducted following a previously described protocol [19]. To prepare serum samples, freeze-preserved sera were thawed at 4°C, vortexed, and centrifuged (825 *g*, 3 min at 4°C). Then, 200 μL of serum was mixed with 200 μL of 0.1 M PBS solution, vortexed, and centrifuged (825 *g*, 3 min at 4°C). Next, precisely 400 μL of the resulting mixture was transferred to 5-mm NMR tubes for subsequent NMR experiments. An internal tube containing 1 mM sodium 3-(trimethylsilyl) propionate-2,2,3,3-d4 (TSP) was used as an internal standard sample for chemical shift calibration and quantitative measurement of metabolite concentration during the experiment.

### NMR spectroscopy

NMR experiments were performed at 298 K using a Bruker AVANCE III HD 850 MHz spectrometer (Bruker BioSpin GmbH, Rheinstetten, Germany) equipped with a TCI cryoprobe. One-dimensional (1D) ^1^H-spectra were recorded on mouse serum samples using the Carr-Purcell-Meiboom-Gill (CPMG) pulse sequence [RD-90°-(τ-180°-τ) n-ACQ]. A total of 64 transients were collected into 64 K data points, with a spectral width of 20 ppm and an acquisition time (ACQ) of 2.73 s. An additional relaxation delay of 4 s was used.

Resonances of metabolites were assigned based on the 1D ^1^H-spectra using a combination of Chenomx NMR Suite software (version 8.3; Chenomx, Edmonton, Canada), the Human Metabolome Data Base (HMDB; http://www.hmdb.ca/), and relevant published references [ 20‒ 22 ]. To confirm the assigned metabolites, selected serum samples were used to record two-dimensional (2D) ^1^H- ^13^C heteronuclear single quantum coherence (HSQC) and ^1^H- ^1^H total correlation spectroscopy (TOCSY) spectra. The relative concentrations of the metabolites were quantified using the integrals of the metabolites normalized to the integral of TSP as an internal reference.

### Metabolomic analysis

To identify metabolic differences between the groups, two metabolomics techniques, principal component analysis (PCA) and orthogonal partial least squares discriminant analysis (OPLS-DA),were performed using SIMCA 14.0 software (MKS Umetrics, Umeå, Sweden). PCA, as an unsupervised method, reduces the complexity of the data by identifying the main sources of variation and grouping the samples based on their similarities. On the other hand, OPLS-DA as a supervised method maximizes the separation between groups while minimizing the variation within groups. The OPLS-DA models used the variable importance in projection (VIP) score to reflect the contribution of each metabolite to the observed metabolic differences between groups. Metabolites with VIP>1 were identified as significant metabolites.

To identify metabolic pathways that were significantly altered between groups under the given conditions, metabolic pathway analysis was performed based on the concentrations of a set of metabolites using the MetaboAnalyst 5.0 web server ( http://www.MetaboAnalyst.ca). The pathway impact value (PIV) reflects the impact of each metabolite on a specific metabolic pathway. Here, metabolic pathways with *P*<0.05 and PIV>0.1 were identified as significant pathways, which may provide insights into the underlying metabolic mechanisms associated with the observed metabolic differences between the groups.

### Statistical analysis

Data are presented as the mean±SD. To quantitatively compare the three groups, one-way ANOVA followed by Tukey’s multiple comparison test was employed using SPSS 22.0 software (IBM, Chicago, USA). The statistical significance was determined as follows: *P*>0.05 (ns), *P*<0.05 (*), *P*<0.01 (**), *P*<0.001 (***), and *P*<0.0001 (****). Metabolites with *P*<0.05 were identified as differential metabolites, whereas metabolites with VIP>1 and *P*<0.05 were recognized as characteristic metabolites.

### Multivariate ROC analysis

Multivariate receiver operating characteristic (ROC) analysis of mouse serum samples was conducted to assess the relevance of metabolites involved in significantly altered metabolic pathways. The logistic regression algorithm was used to construct ROC curves with MetaboAnalyst 5.0. To identify potential biomarkers for predicting CAC stage from NCAC stage, we combined the results of univariate and multivariate analyses with the ROC analysis of the metabolites. The criteria for selecting potential biomarkers were a change in metabolite concentration Log _2_ (fold change)>0.6 or <‒1, VIP>1, and an area under the curve (AUC)>0.7. This approach allowed us to identify the most relevant metabolites for distinguishing between the CAC and NCAC stages.

## Results

### Characterization of the progression of CAC in mice

**Figure 1:**
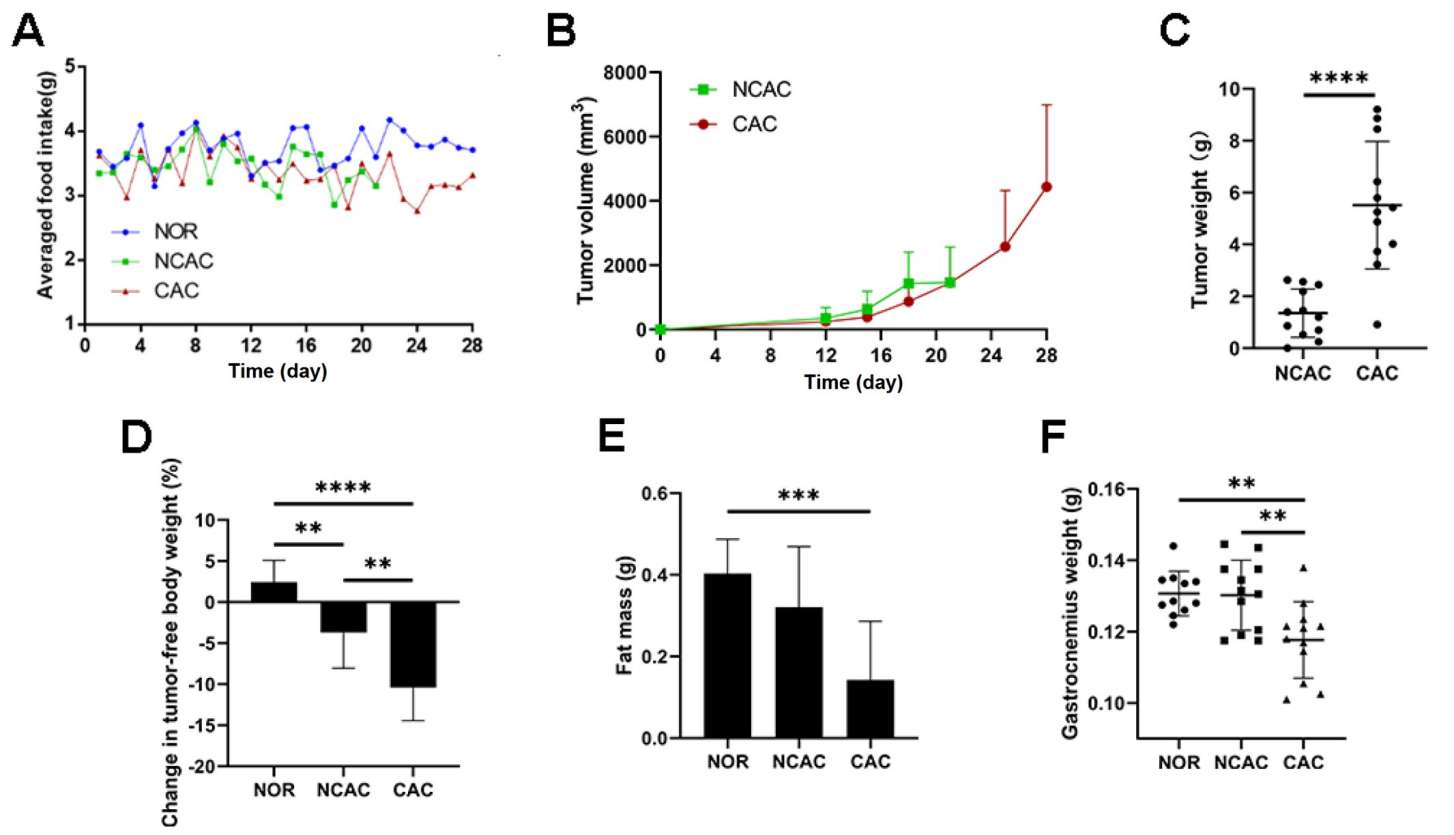
Figure 1 Characterization of a mouse model of colon CAC (A) Average daily food intake curves of NOR, NCAC, and CAC mice. (B) Tumor growth curves of NCAC and CAC mice, the tumor size is expressed as tumor volume (mm3 )=0.52×length×width2. (C) Tumor weights of CAC and NCAC mice at the time of sacrifice. (D) Percentage changes in tumor-free body weight of the mice relative to the body weight at the time of initial animal modeling. (E) Weights of epididymal adipose tissue of mice. (F) Weights of the gastrocnemius muscle of hind limbs of the mice. **P<0.01, ***P<0.001, ****P<0.0001.

In this study, we observed changes in physical conditions (including tumor growth, weight changes, and behavioral characteristics) during the progression of colon CAC in mice. On day 7, the mice developed tumors on the right flank, which grew to the size of a grain of rice. On day 21, mice that had developed tumors with insignificant body weight loss (<5% of their initial body weight) showed no signs of emaciation, anorexia, or poor activity. On day 28, anorexia symptoms were observed in the CAC mice, while both tumor weight and volume rapidly increased during the CAC stage ( Figure 1A‒C). To determine the severity of cachexia, the ratio of tumor-free body weight to the corresponding baseline body weight was calculated. The results showed that cachexic mice experienced a body weight loss of over 5% ( Figure 1D), whereas noncachexic mice experienced a body weight loss of less than 5%. Cancer cachexia in mice was well characterized by the loss of both fat and skeletal muscle ( Figure 1E,F). We did not observe any tumor metastasis in the euthanized mice.

**Figure 2:**
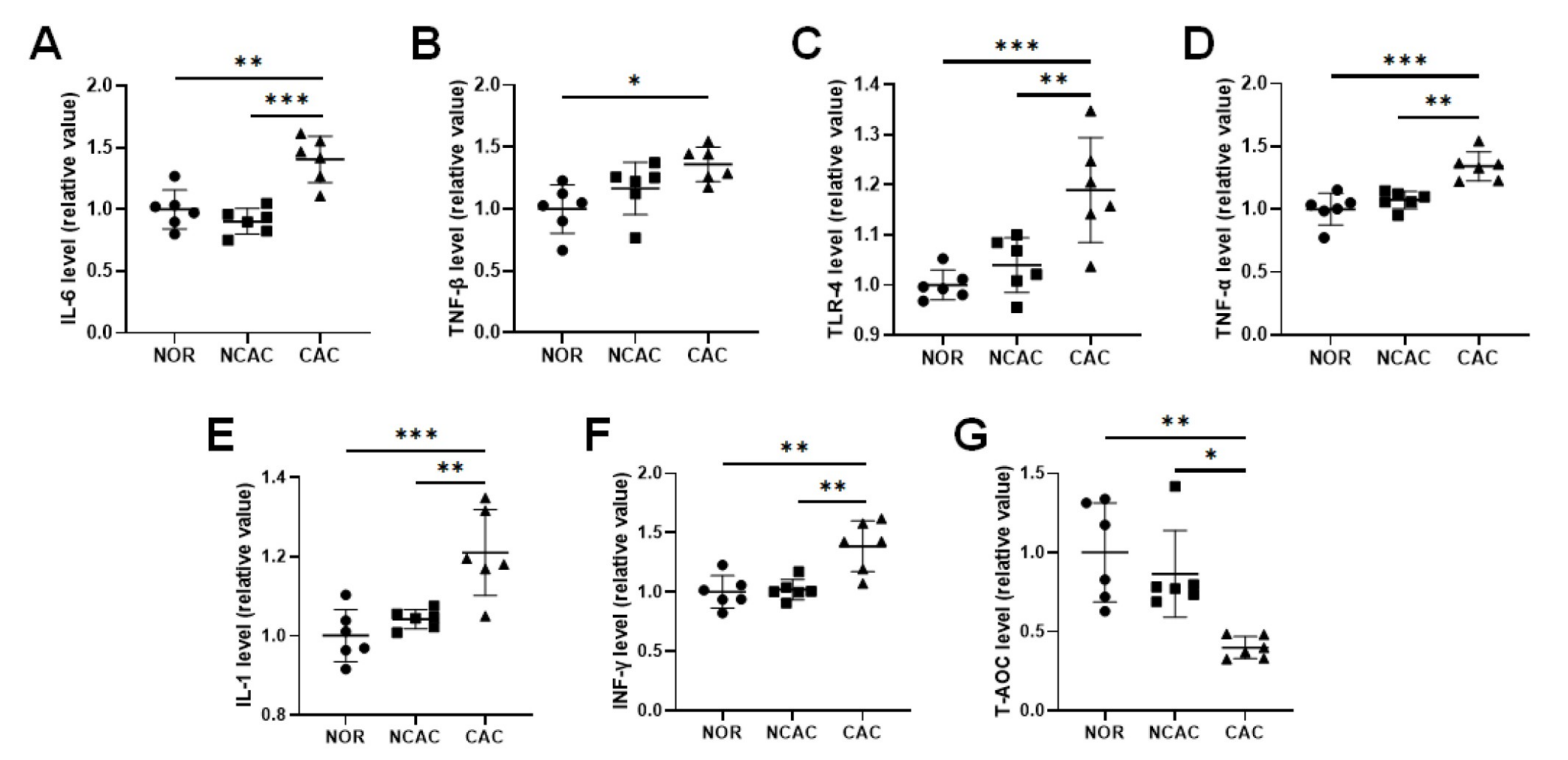
Figure 2 Levels of inflammatory factors in the three groups of mouse sera (A) IL-1. (B) IL-6. (C) TLR-4. (D) TNF-α. (E) TGF-β. (F) IFN-γ. (G) Total antioxidant capacity (T-AOC). *P<0.05, **P<0.01, ***P <0.001.

The presence of systemic inflammation is commonly associated with CAC. In our animal experiments, we observed a significant increase in serum inflammatory factors, including IL-1, IL-6, TLR-4, TNF-α, TGF-β, and IFN-γ, in CAC mice compared to NCAC mice ( Figure 2A‒F). Additionally, cachexia induced oxidative stress, as evidenced by a notable decrease in the total antioxidant capacity in CAC mice compared to both NCAC and NOR mice ( Figure 2G). Surprisingly, no noticeable changes in these serum inflammatory factors or the total antioxidant capacity were observed in the NCAC mice compared to the normal controls ( Figure 2). These findings highlight the distinct differences in oxidative stress between the CAC and NCAC stages.

### NMR spectra of sera and multivariate data analysis

**Figure 3:**
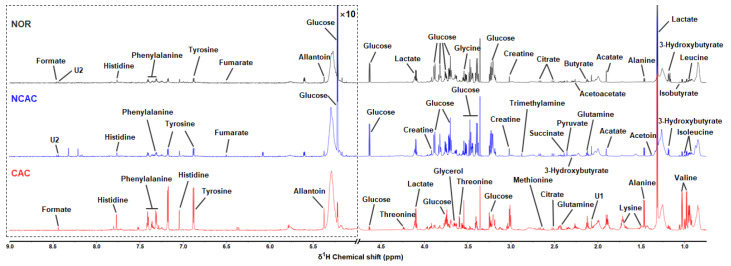
Figure 3 Typical 1D ^1^H-NMR spectra of sera from the three groups of mice The spectra were recorded on a Bruker Avance III 850 MHz NMR spectrometer at 298 K (pH 7.4). Spectral regions of 0.5‒4.7 ppm and 5.0‒9.0 ppm are displayed, and the water region of 4.7–5.0 ppm was removed. The region of 5.0‒9.0 ppm has been magnified 10 folds compared to another region of 0.5‒4.7 ppm for clarity. Assigned metabolites are labeled in this figure and shown in Supplementary Table S1.

Typical 850 MHz 1D ^1^H-NMR spectra were recorded on aqueous metabolites from the NOR, NCAC, and CAC groups of mouse sera ( Figure 3). The resonances of 31 metabolites, including 2 unknown metabolites, were assigned and confirmed by 2D ^1^H- ^13^C HSQC and ^1^H- ^1^H TOCSY spectra ( Supplementary Figure S2). These assigned metabolites are listed in Supplementary Table S1.

**Figure 4:**
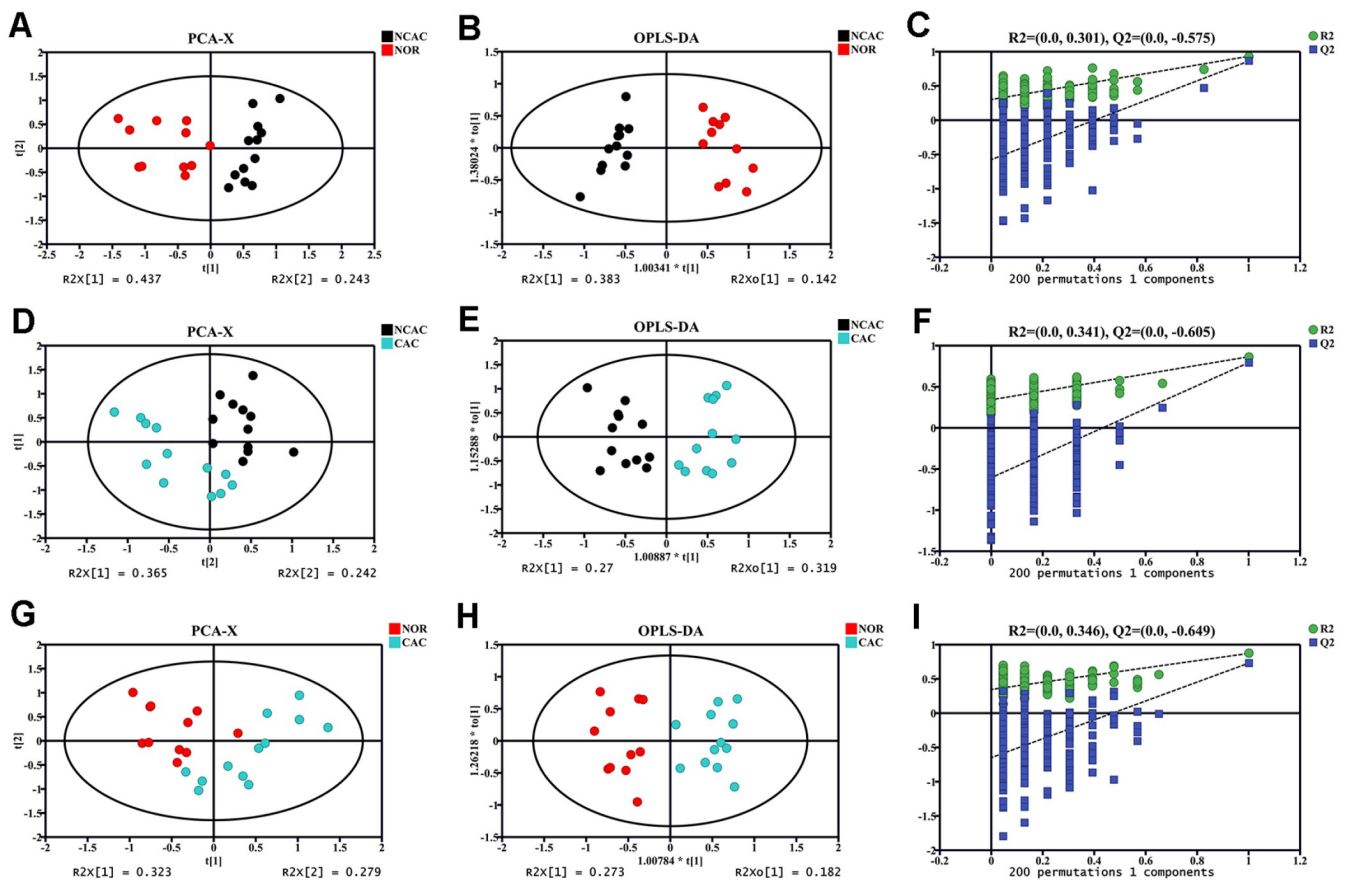
Figure 4 Scores plots and cross-validation plots of PCA and OPLS-DA models based on 1D ^1^H-NMR spectral data of the three groups of mouse sera (A‒C) NCAC vs NOR. (D‒F) CAC vs NCAC. (G‒I) CAC vs NOR. The ellipses indicate the 95% confidence limit. Random permutation test was performed to evaluate the reliability of the OPLS-DA model with 200 cycles.

Unsupervised PCA analyses were performed on the NMR datasets of the three groups of mouse sera. The PCA score plots indicate distinct metabolic profiles for the NOR, NCAC and CAC groups ( Figure 4A,D,G). Subsequently, supervised OPLS-DA analyses were conducted on the NMR datasets to enhance the metabolic differences among the three groups. The OPLS-DA score plots demonstrate clear separations between NCAC and NOR, CAC and NCAC, and CAC and NOR ( Figure 4B,E,H). The reliability of the OPLS-DA models was verified using a response permutation test (RPT) with 200 cycles ( Figure 4C,F,I). The obtained R ^2^Y and Q ^2^Y values for the three OPLS-DA models were as follows: R ^2^Y=0.932 and Q ^2^Y=0.861 for NCAC vs NOR; R ^2^Y=0.865 and Q ^2^Y=0.793 for CAC vs NCAC; and R ^2^Y=0.872 and Q ^2^Y=0.729 for CAC vs NOR.

### Identifications of differential, significant and characteristic metabolites

Metabolite integrals normalized to the TSP integral were used to express the relative concentrations of metabolites. To quantitatively compare the relative concentrations of metabolites among the three groups of mouse sera, one-way ANOVA was conducted. Using the criteria of *P*<0.05, differential metabolites were identified from the pairwise comparisons between the three groups ( Table 1): 14 differential metabolites for NCAC vs NOR, including isoleucine, isobutyrate, acetoin, acetate, acetoacetate, 3-hydroxybutyrate, pyruvate, succinate, trimethylamine, creatine, glycerol, glycine, threonine, and glucose; 16 differential metabolites for CAC vs NCAC, including leucine, isoleucine, isobutyrate, acetoin, lysine, acetate, pyruvate, citrate, methionine, trimethylamine, creatine, lactate, glucose, allantoin, phenylalanine, and U2; and 17 differential metabolites for CAC vs NOR, including leucine, isobutyrate, alanine, lysine, acetate, acetoacetate, 3-hydroxybutyrate, succinate, citrate, methionine, trimethylamine, glycerol, threonine, allantoin, tyrosine, phenylalanine, and U2.

**Table:** **Table 1** Quantitative comparisons of relative concentrations of metabolites calculated from 1D ^1^H-NMR spectra of mouse sera

Metabolite	Mean±SD		Multiple comparison		One-way ANOVA
	NOR	NCAC	CAC		NCAC vs NOR	CAC vs NCAC	CAC vs NOR		F	*P*
Leucine	0.118±0.021	0.117±0.016	0.150±0.046		ns	**↑**	**↑**		4.191	0.024
Isoleucine	0.095±0.021	0.073±0.012	0.096±0.026		**↓**	**↑↑**	ns		4.579	0.018
Valine	0.232±0.034	0.186±0.038	0.223±0.118		ns	ns	ns		1.199	0.314
Isobutyrate	0.056±0.020	0.035±0.011	0.076±0.020		**↓↓**	**↑↑↑↑**	**↑**		15.863	<0.001
Acetoin	0.120±0.017	0.081±0.035	0.120±0.021		**↓↓↓**	**↑↑↑**	ns		9.718	<0.001
Alanine	0.329±0.029	0.392±0.062	0.465±0.144		ns	ns	**↑↑**		6.102	0.006
Lysine	0.162±0.021	0.144±0.031	0.240±0.078		ns	**↑↑↑↑**	**↑↑↑**		12.360	<0.001
Acetate	0.416±0.148	0.124±0.032	0.219±0.080		**↓↓↓↓**	**↑**	**↓↓↓↓**		27.148	<0.001
Butyrate	0.123±0.019	0.114±0.014	0.121±0.019		ns	ns	ns		0.831	0.445
Acetoacetate	0.138±0.031	0.073±0.011	0.093±0.029		**↓↓↓↓**	ns	**↓↓↓**		19.965	<0.001
3-Hydroxybutyrate	0.097±0.025	0.048±0.011	0.052±0.016		**↓↓↓↓**	ns	**↓↓↓↓**		25.623	<0.001
Pyruvate	0.116±0.020	0.156±0.028	0.124±0.026		**↑↑**	**↓↓**	ns		8.251	0.001
Succinate	0.119±0.016	0.058±0.013	0.055±0.020		**↓↓↓↓**	ns	**↓↓↓↓**		56.356	<0.001
Glutamine	0.229±0.031	0.213±0.020	0.215±0.046		ns	ns	ns		0.757	0.477
Citrate	0.095±0.010	0.084±0.011	0.127±0.031		ns	**↑↑↑↑**	**↑↑**		14.482	<0.001
Methionine	0.073±0.017	0.075±0.019	0.100±0.027		ns	**↑↑**	**↑↑**		6.169	0.005
Trimethylamine	0.021±0.005	0.055±0.012	0.039±0.024		**↑↑↑↑**	↓	**↑**		11.877	<0.001
Creatine	0.218±0.034	0.176±0.009	0.230±0.045		**↓↓**	**↑↑↑**	ns		8.738	<0.001
Glycerol	0.217±0.025	0.161±0.016	0.155±0.029		**↓↓↓↓**	ns	**↓↓↓↓**		22.374	<0.001
Glycine	0.379±0.040	0.323±0.036	0.339±0.053		**↓**	ns	ns		4.738	0.016
Threonine	0.208±0.017	0.187±0.017	0.189±0.022		**↓**	ns	**↓**		4.479	0.019
Lactate	1.418±0.215	1.510±0.354	1.207±0.319		ns	↓	ns		3.262	0.051
Glucose	0.777±0.180	1.032±0.083	0.848±0.098		**↑↑↑↑**	**↓↓↓**	ns		12.997	<0.001
Allantoin	0.022±0.005	0.019±0.003	0.034±0.006		ns	**↑↑↑↑**	**↑**		28.338	<0.001
Fumarate	0.003±0.002	0.003±0.001	0.003±0.002		ns	ns	ns		1.068	0.355
Tyrosine	0.034±0.006	0.040±0.010	0.047±0.015		ns	ns	**↑**		3.519	0.041
Phenylalanine	0.040±0.005	0.041±0.003	0.049±0.013		ns	**↑**	**↑**		3.911	0.030
Histidine	0.013±0.003	0.014±0.002	0.018±0.009		ns	ns	ns		1.853	0.173
Formate	0.001±0.001	0.001±0.001	0.001±0.001		ns	ns	ns		0.812	0.453
U1	0.204±0.011	0.212±0.012	0.208±0.013		ns	ns	ns		1.299	0.286
U2	0.003±0.001	0.005±0.001	0.007±0.003		ns	**↑↑**	**↑↑↑↑**		11.418	<0.001

Statistical significance was represented by *P*-value determined by one-way ANOVA followed by Tukey’s multiple comparison tests: ns, *P*>0.05; ↑/↓, *P*<0.05; ↑↑/↓↓, *P*<0.01; ↑↑↑/↓↓↓, *P*<0.001; ↑↑↑↑/↓↓↓↓, *P*<0.0001. Differential metabolites were identified with *P*<0.05. The upward and downward arrows denote that the difference between A and B is positive (A is increased compared to B) and negative (A is decreased compared to B), respectively.

**Figure 5:**
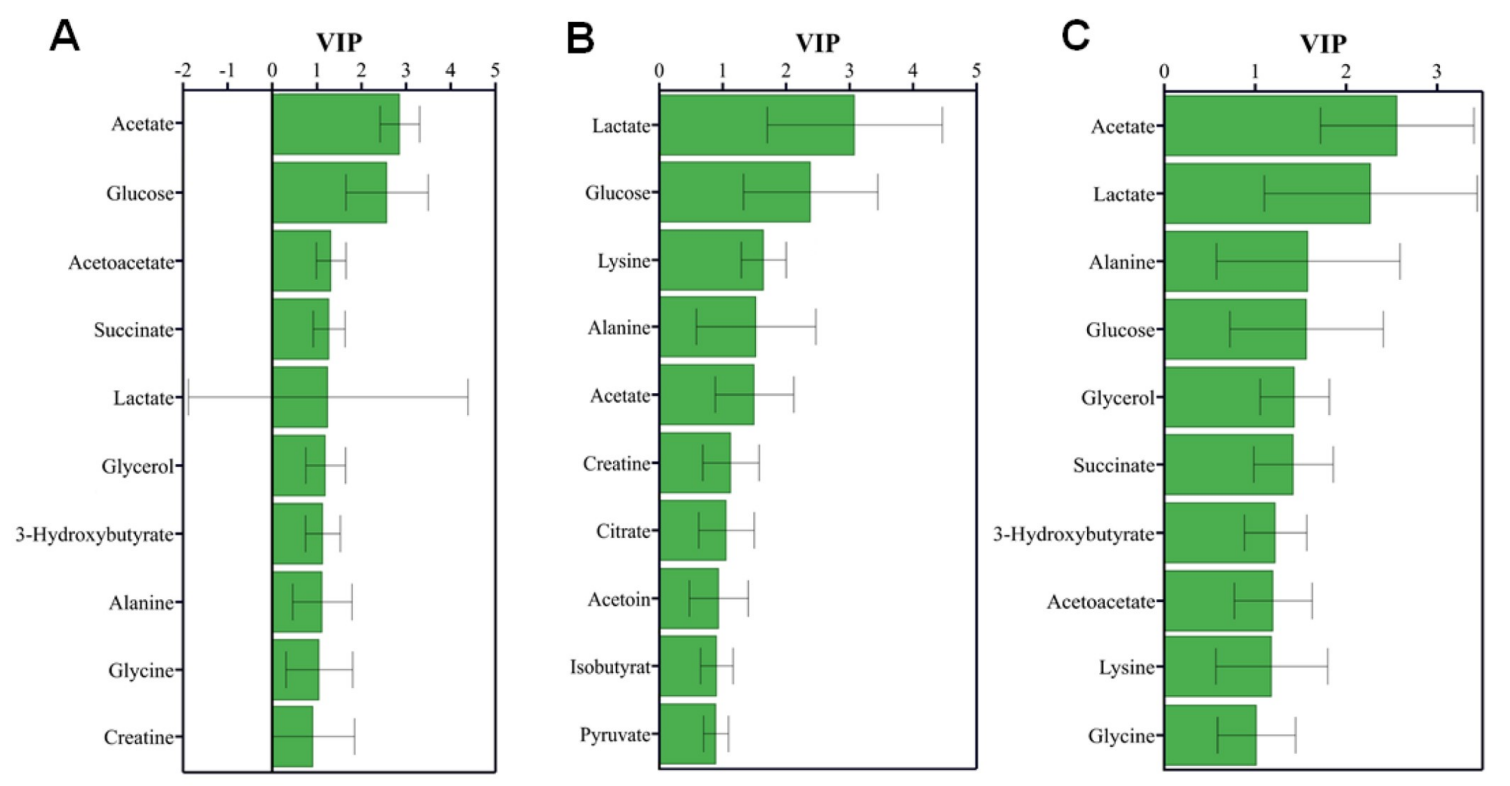
Figure 5 VIP score-ranking significant metabolites identified from the three OPLS-DA models of mouse sera These VIP scores were obtained from pair-wise comparisons of (A) NCAC vs NOR, (B) CAC vs NCAC, and (C) CAC vs NOR. Significant metabolites were identified with VIP>1.

Using the criteria of VIP>1, significant metabolites were identified from the established OPLS-DA models of mouse sera ( Figure 5): 9 metabolites for NCAC vs NOR, including acetate, glucose, acetoacetate, succinate, lactate, glycerol, 3-hydroxybutyrate, alanine, glycine; 7 metabolites for CAC vs NCAC, including lactate, glucose, lysine, alanine, acetate, creatine, citrate; 10 metabolites for CAC vs NOR, including acetate, lactate, alanine, glucose, glycerol, succinate, 3-hydroxybutyrate, acetoacetate, lysine, glycine.

Using two criteria of VIP>1 and *P*<0.05, characteristic metabolites were identified ( Table 2): 7 metabolites for NCAC vs NOR, including glucose, acetate, acetoacetate, succinate, glycerol, 3-hydroxybutyrate, and glycine; 6 metabolites for CAC vs NCAC, including lactate, glucose, lysine, acetate, creatine, and citrate; and 7 metabolites for NCAC vs NOR, including acetate, alanine, glycerol, succinate, 3-hydroxybutyrate, acetoacetate, and lysine.

**Table:** **Table 2** Characteristic metabolites identified through pairwise comparisons among the three groups of mouse sera

Metabolite	NCAC vs NOR	CAC vs NCAC	CAC vs NOR
Lactate		↓	
Glucose	**↑↑↑↑**	**↓↓↓**	
Lysine		**↑↑↑↑**	**↑↑↑**
Alanine			**↑↑**
Acetate	**↓↓↓↓**	**↑**	**↓↓↓↓**
Creatine		**↑↑↑**	
Citrate		**↑↑↑↑**	
Glycerol	**↓↓↓↓**		**↓↓↓↓**
Succinate	**↓↓↓↓**		**↓↓↓↓**
3-Hydroxybutyrate	**↓↓↓↓**		**↓↓↓↓**
Acetoacetate	**↓↓↓↓**		**↓↓↓**
Glycine	**↓**		

Characteristic metabolites were determined by a combination of the significant metabolites identified from the OPLS-DA models (VIP>1) and differential metabolites identified from the univariate analyses ( *P*<0.05). The upward and downward arrows denote that the difference between A and B is positive (A is increased compared to B) and negative (A is decreased compared to B), respectively.

### Significant metabolic pathways during the progression of CAC

**Figure 6:**
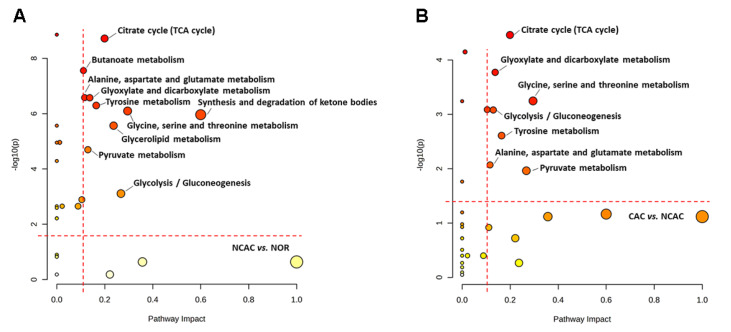
Figure 6 Metabolic pathway analysis for the three groups of mouse sera (A) NCAC vs NOR. (B) CAC vs NCAC. Significantly altered metabolic pathways were identified with pathway impact value (PIV)>0.1 and P<0.05, using the pathway analysis module provided by MetaboAnalyst 5.0 webserver.

To identify significantly altered metabolic pathways (significant pathways), we performed metabolic pathway analysis using two criteria: PIV>0.1 and *P*<0.05 ( Figure 6 and Supplementary Table S2). The two comparisons of NCAC vs NOR and CAC vs NCAC revealed 10 and 7 significant pathways, respectively. Notably, 6 significant pathways are shared by the three comparisons, including the TCA cycle; alanine, aspartate, and glutamate metabolism; glyoxylate and dicarboxylate metabolism; tyrosine metabolism; glycolysis/gluconeogenesis; and pyruvate metabolism. These metabolic pathways are commonly impaired during the progression of CAC.

### Potential biomarkers for differentiating between CAC and NCAC stages

**Figure 7:**
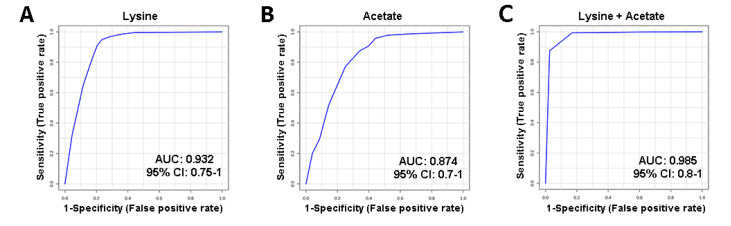
Figure 7 Multivariate ROC analysis based on serum levels of differential metabolites for distinguishing between CAC and NCAC stages (A) Lysine. (B) Acetate. (C) Combination of lysine and acetate.

To identify potential biomarkers for differentiating CAC mice from NCAC mice, we performed ROC analysis based on the relative concentrations of differential metabolites between the two groups ( Table 3). Two characteristic metabolites, lysine and acetate, were identified as potential biomarkers with AUC values of 0.932 and 0.874, respectively ( Figure 7A,B). The combination of lysine and acetate showed the highest AUC value of 0.985 ( Figure 7C). These results suggest that lysine and acetate may serve as potential biomarkers for distinguishing between cachexia and noncachexia mice.

**Table:** **Table 3** Potential biomarkers for distinguishing between the CAC and NCAC stages

Metabolite	ROC analysis		Univariate & multivariate analysis
	AUC	*P*	Log _2_ (fold change)		Significance	VIP
Isobutyrate	0.981	<0.001	1.098		↑↑↑↑	0.906
Allantoin	0.974	<0.001	0.825		↑↑↑↑	0.659
Lysine	0.942	0.001	0.739		↑↑↑↑	1.645
Glucose	0.942	<0.001	‒0.284		↓↓↓	2.385
Citrate	0.885	<0.001	0.596		↑↑↑↑	1.057
Creatine	0.885	<0.001	0.386		↑↑↑	1.129
Acetate	0.859	0.001	0.824		↑	1.499
Pyruvate	0.833	0.008	‒0.327		↓↓	0.893
Leucine	0.814	0.031	0.350		↑	0.717
Acetoin	0.814	0.002	0.580		↑↑↑	0.938
U2	0.788	0.011	0.616		↑↑	0.225
Isoleucine	0.776	0.011	0.382		↑↑	0.656
Methionine	0.776	0.012	0.420		↑↑	0.839
Trimethylamine	0.769	0.050	‒0.501		↓	0.397
Lactate	0.750	0.034	‒0.323		↓	3.080
Acetoacetate	0.731	0.033	0.356		ns	0.497
Fumarate	0.718	0.138	‒0.418		ns	0.124

Criteria for screening potential biomarkers: high AUC values (AUC>0.7), significant changes in serum metabolite concentration [Log _2_(fold change)>0.6 or <‒1], and large variable importance projection (VIP>1) during the transition from NCAC to CAC.

## Discussion

CAC is a devastating syndrome that significantly contributes to the morbidity and mortality of cancer patients. Deciphering the metabolic alterations and underlying molecular mechanisms underlying the progression of CAC is crucial for the diagnosis and effective management of this syndrome. Despite the proposed guidelines for classifying CAC [3], assessing its progression remains a challenge for clinicians. Monitoring body weight loss alone is insufficient and lacks sensitivity and specificity. Therefore, there is an urgent need to develop sensitive, simple, and reliable indicators for assessing cachexia progression. Such indicators would enable clinicians to accurately stage cachexia in cancer patients and tailor personalized treatment strategies to their unique need.

In this study, we established a mouse model of colon CAC, analyzed metabolic signatures during cachexia progression, and identified potential biomarkers for diagnosing cachexia development. We categorized these mice into three groups: NCAC, CAC and NOR groups based on changes in body weight. CAC mice exhibited typical cachexia characteristics [23], such as body weight loss, skeletal muscle atrophy, and anorexia symptoms, while NCAC mice did not. We observed significant metabolic differences between CAC and NCAC mice and found that cachexia upregulated the levels of inflammatory factors and decreased the total antioxidant capacity, indicating the existence of oxidative stress. These changes are associated with the secretion of various inflammatory factors by tumor cells during cachexia development, resulting in inflammatory reactions in different organs and systemic circulation, leading to metabolic disorders [24].

Metabolic pathway analysis showed significant impairments in energy metabolism ( *e*. *g*., TCA cycle, glycolysis/gluconeogenesis, pyruvate metabolism) and amino acid metabolism ( *e*. *g*., alanine, aspartate and glutamate metabolism, tyrosine metabolism, and glycine, serine and threonine metabolism) during cachexia progression ( Figure 6 and Supplementary Table S2). We also observed significant differences in the concentrations of several metabolites involved in energy metabolism, such as pyruvate, succinate, citrate, acetate, glucose, and lactate, indicating that cachexia impairs energy metabolism by causing an imbalance between energy intake and utilization. The impairment in energy metabolism contributes significantly to the onset and development of cachexia, as shown in previous studies [ 3, 25‒ 27]. Cachexia has been shown to be associated with impaired energy metabolism in a variety of cancers, including pancreatic cancer [28], breast cancer [29], ovarian cancer [30], and kidney cancer [31]. However, due to the different metabolic characteristics of cancer patients at different stages of cachexia, the efficiency of energy metabolism may be different in cancer patients [32]. Therefore, identifying biomarkers that can differentiate between noncachexic and cachexic stages in cancer patients is crucial for developing personalized treatment strategies.

The progression of CAC is associated with significant impairments in amino acid metabolism, in addition to impairments in energy metabolism. During the transition from normal to noncachexia, most free amino acids, particularly BCAAs, were downregulated, while during the progression from noncachexia to cachexia, most amino acids were upregulated ( Table 1). Cachexia induces substantial metabolic changes in the body, affecting amino acids, carbohydrates, and lipids, which may ultimately contributes to tumor growth [ 33‒ 35]. In the noncachexia stage, the body rapidly responds to the disease stimulus by providing free amino acids in the bloodstream to other organs for stress repair. This response aims to balance protein synthesis and skeletal muscle degradation, ultimately reducing muscle loss and maintaining muscle mass. In the cachexia stage, however, severe muscle loss leads to skeletal muscle protein degradation, an increase in myogenin, and the production of amino acids by skeletal muscle via the TCA cycle, which are released into the circulation and may provide metabolic substrates for tumors. The dynamics of amino acids during the development of cachexia ultimately contributes to the negative nitrogen balance of muscle [ 36‒ 38]. These results suggest that amino acid metabolism plays a critical role in the progression of cachexia, and identifying biomarkers relevant to amino acid metabolism may help differentiate between noncachexic and cachexic stages in cancer patients.

To distinguish between cachexia and noncachexia stages, we identified potential biomarkers using a combination of ROC, univariate, and multivariate analyses. Our selection criteria included metabolites with significant concentration changes, large VIP scores, and high AUC values during the progression from NCAC to CAC. Lysine and acetate emerged as potential biomarkers for differentiating CAC mice from NCAC mice. Specifically, serum levels of lysine and acetate were significantly increased during the progression from noncachexia to cachexia, reflecting alterations in carbohydrate and amino acid metabolism ( Table 3). The combination of lysine and acetate showed a high AUC value of 0.991, indicating its excellent ability to differentiate the CAC stage from the NCAC stage ( Figure 7). Our findings suggest that these two metabolites hold promise as potential biomarkers for monitoring the progression of cachexia in cancer patients. In addition, we assessed the ability of acetate and lysine to distinguish NCAC mice from NOR mice and found that acetate could serve as a potential biomarker for distinguishing the NCAC stage from the NOR stage with a high AUC value of 0.931, but lysine could not, as shown in Supplementary Figure S3.

Acetate, like glucose, serves as an energy substrate in mammals and is converted into acetyl-CoA for energy and lipid metabolism [ 39, 40]. Presently, acetate has emerged as an alternative energy source for many cancer cells, serving as the primary source of acetyl-CoA under stress conditions [ 41‒ 43], including glioblastoma [44], liver cancer [45], and colorectal cancer [46]. In a metabolomic analysis of sera from metastatic breast cancer patients, Jiang *et al*. [47] found significantly lower acetate concentrations during breast cancer progression, suggesting acetate as a potential biomarker for disease progression. Similarly, Gupta *et al*. [48] reported increased acetate levels in squamous oral carcinoma patients, proposing acetate as a biomarker for distinguishing oral squamous leukoplakia from squamous oral carcinoma. In lung cancer patients, Singh *et al*. [49] demonstrated elevated serum acetate levels using NMR technology, indicating acetate as a biomarker for lung cancer diagnosis. In a previous study, Gu *et al*. [50] found higher serum acetate concentrations in colon polyp and colon cancer patients than in normal controls, with the serum acetate/glycerol ratio being proposed as an early biomarker for colon cancer development from colon polyps.

Consistent with previous studies, we found that serum acetate level increased during the transition from the NCAC to CAC stages. In the noncachexia stage, tumor cell growth is in its initial phase, and acetate is utilized by tumor tissues to provide energy, while glucose is trapped at a high level due to chronic inflammation and insulin resistance [ 51, 52]. In the cachexia stage, however, the growth and metabolism of cancer cells are vigorous, and the energy demand of tumor tissues is high. Glucose is utilized as the main energy supply substrate, and the serum glucose level decreases [ 53, 54]. The elevation of serum acetate level in the CAC stage may be related to the conversion of energy supply substrates and energy demand across different stages of cancer cachexia, where acetate may serve as an alternative energy source for cancer cells during periods of stress and high energy demand. Therefore, serum acetate level may serve as a potential biomarker for the progression and severity of cachexia in cancer patients.

The elevated serum lysine level observed in cachexia may be attributed to impairments in amino acid metabolism and the breakdown of muscle mass. Lysine, as an essential amino acid, plays a critical role in protein synthesis [55]. Several studies have consistently reported increased serum lysine level during cachexia. For instance, Yang *et al* . [56] used NMR-based serum metabolomics to analyze different stages of cachexia and observed an increase in serum lysine content from precachexia to cachexia. Similarly, Peters *et al* . [57] reported a 204% increase in serum lysine content in cancer cachexia mice. Despite the lack of identification of lysine as a biomarker for different stages of CAC in previous research, this could be due to the confusion between different stages of cachexia. Nevertheless, changes in lysine level may correspond to impairments in amino acid metabolism, as reported above in our pathway analysis. Our results suggest that lysine may serve as a promising biomarker to distinguish between NCAC and CAC stages.

In recent years, many studies have reported potential biomarkers for the diagnosis of cachexia, such as MCP-1 [58], GDF-15 [59], and IL-6 [60]. However, most of these biomarkers are not applicable to colon cancer cachexia. In the present study, we identified acetate and lysine as potential combined biomarkers with a high AUC value of 0.985, which was higher than those of the above-described biomarkers with AUC values ranging from 0.700 to 0.950, suggesting that the combination of lysine and acetate as potential biomarkers may be more effective in distinguishing the cachexia stage of colon cancer from the noncachexia stage.

Our study highlights the dynamic changes in serum levels of lysine and acetate during the progression of colon CAC, underscoring their potential as biomarkers for differentiating between noncachexia and cachexia stages. The combination of these two metabolites shows promise for the diagnosis of colon CAC. However, due to the limited sample size, our findings are based on a training dataset, necessitating further research with a larger number of cancer mice to validate the diagnostic model. Additionally, the clinical applicability of the identified potential biomarkers needs to be evaluated in serum samples. Nonetheless, these biomarkers hold potential in the diagnosis and treatment of CAC, and further research is warranted to assess their clinical utility.

In summary, in this study, we established a mouse model of colon cancer cachexia and employed NMR-based metabonomic profiling along with multivariate ROC analysis on serum samples from CAC mice, NCAC mice, and normal controls. Our findings revealed distinct metabolic signatures associated with the CAC and NCAC stages during the progression of CAC, highlighting the significant role of impaired energy metabolism and amino acid metabolism in cachexia progression. Moreover, we observed elevated serum levels of lysine and acetate during the transition from NCAC to CAC stages, suggesting their potential critical roles in the progression of colon cancer cachexia. Importantly, lysine and acetate emerged as promising serum biomarkers for differentiating between cachexia and noncachexia stages. The robust discriminatory ability of these biomarkers holds great potential for facilitating the diagnosis and treatment of CAC. By identifying these metabolic signatures, our study provides novel insights into the molecular mechanisms underlying the progression of colon CAC. Furthermore, our findings offer valuable avenues for the diagnosis and treatment of CAC in clinical settings, ultimately improving patient outcomes.

## Supporting information

Supplementary

## Supplementary Data

Supplementary data is available at *Acta Biochimica et Biophysica Sinica* online.
