# Supplementary material for: Metabolic signatures and potential biomarkers for the diagnosis and treatment of colon cancer cachexia: NMR metabolomic analysis of serum from cancer cachexia mice
Source: Acta Biochim Biophys Sin (Shanghai). 2023 Sep 14;55(12):1913–24. doi: 10.3724/abbs.2023151 (PMC11294056; doi:10.3724/abbs.2023151)
Supplement: Supplementary [file Supplementary.pdf]

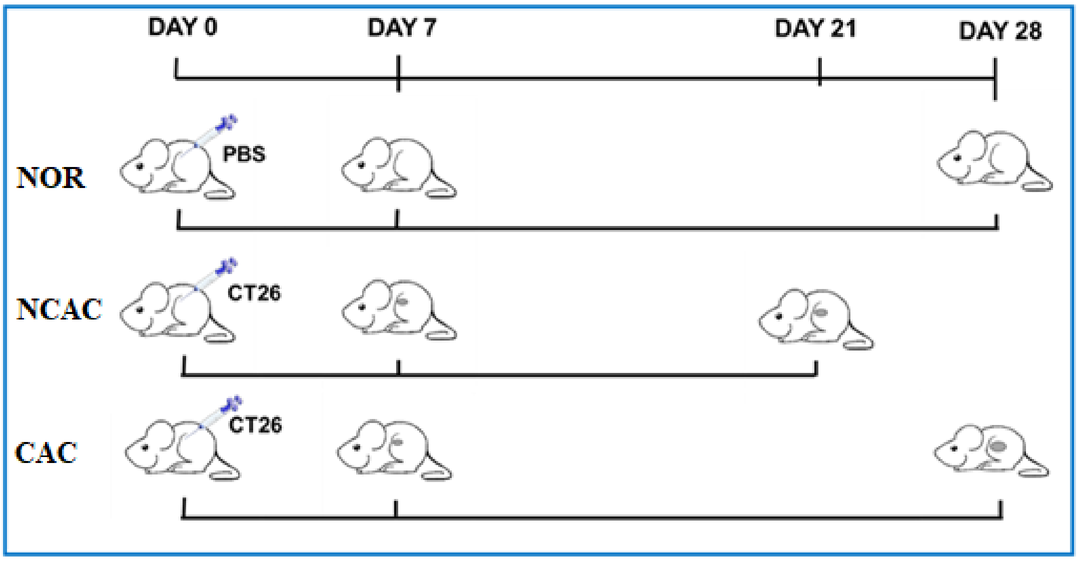

Supplementary Figure S1. Schematic diagram of the animal modeling process At the beginning of the process, some BALB/c mice were inoculated with CT26 cells and defined as tumor mice, while some other BALB/c mice were injected with an equal volume of PBS and defined as normal mice. On day 7, tumors of size of rice grains appeared in the tumor mice. On day 21, the tumor mice developed solid tumors without significant side effects such as slow movement and emaciation, some of which were sacrificed and defined as the NCAC mice. On day 28, some of the tumor mice showing rapid tumor growth, lethargy, slow movement and emaciation were sacrificed and defined as the CAC mice. All NCAC mice were euthanized on day 21, while all CAC and NOR mice were euthanized on day 28.

a.

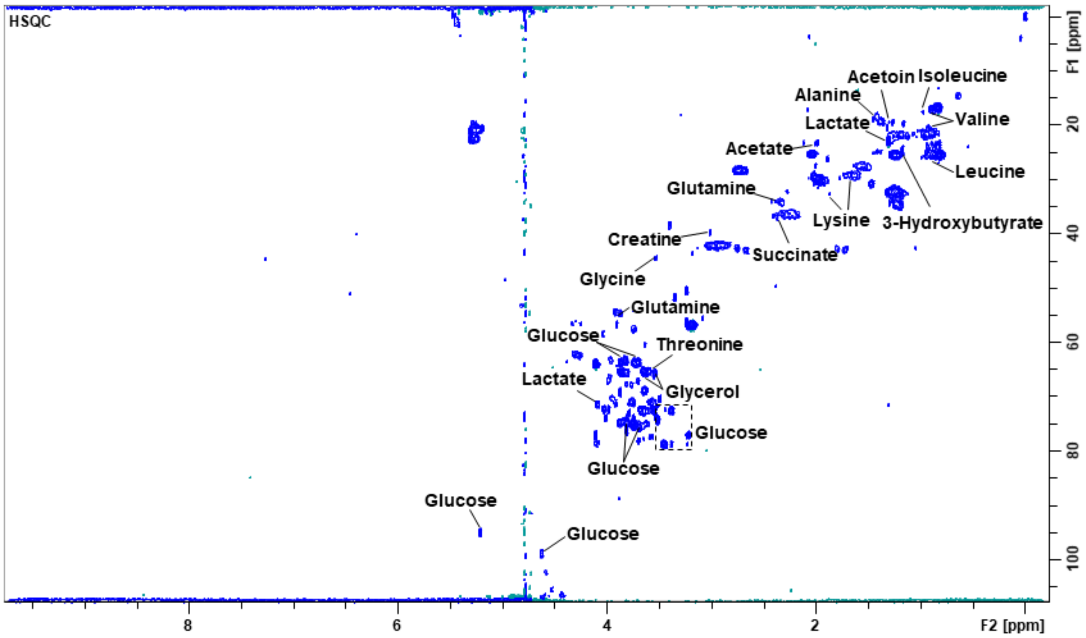

b.

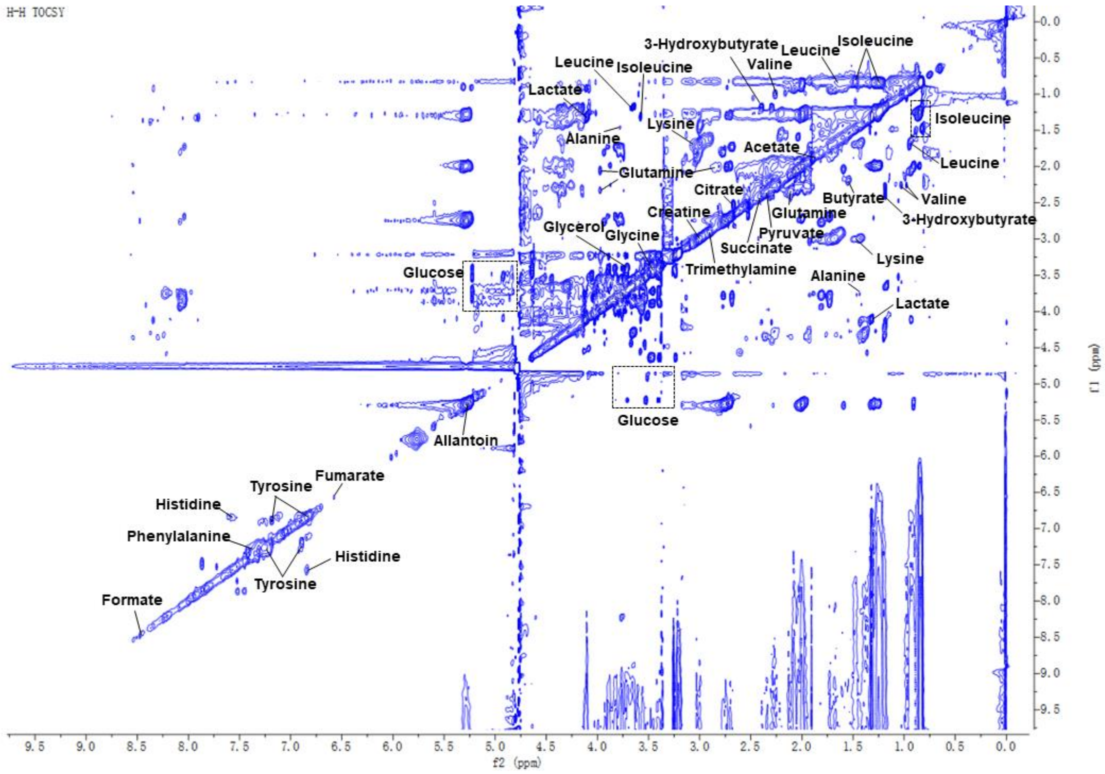

Supplementary Figure S2. 2D ^{1}H-^{13}C-HSQC and ^{1}H-^{1}H-TOCSY spectra of mouse sera (A) ^{1}H-^{13}C-HSQC. (B) ^{1}H-^{1}H-TOCSY. These spectra were recorded on a Bruker Avance III 850 MHz NMR spectrometer at 298 K (pH 7.4). The displayed abbreviations of the metabolite names are given in **Supplementary Table S1**.

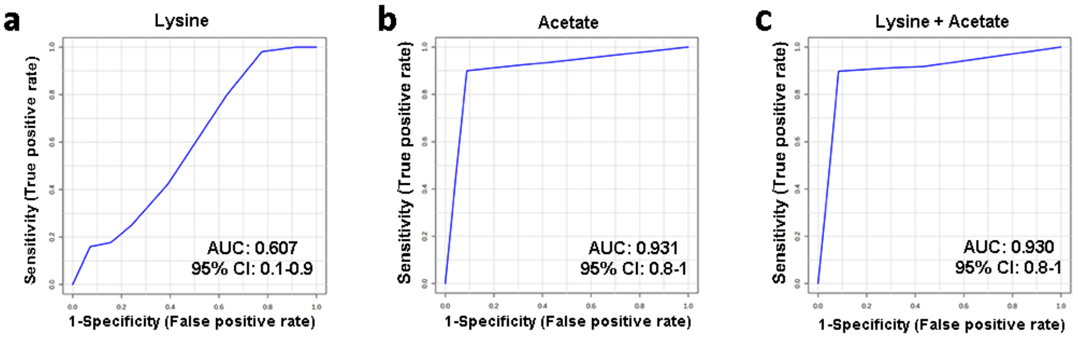

Supplementary Figure S3. Multivariate ROC analysis based on serum levels of two differential metabolites for distinguishing the NCAC stage from the NOR stage (A) Lysine. (B) Acetate. (C) Combination of lysine and acetate.

Supplementary Table S1. Assigned metabolites based on ^{1}H NMR-spectra of mouse sera

No.	Metabolite	δ^1H(ppm) and multiplicity
1	Isoleucine	0.92(t), 0.99(d)
2	Leucine	0.94(d), 0.96(d)
3	Valine	0.97 (d), 1.03(d), 2.27(m)
4	Isobutyrate	1.06 (d)
5	3-Hydroxybutyrate	1.19 (d), 2.28 (dd)
6	Lactate	1.28 (d), 4.10 (q)
7	Threonine	1.30 (d), 3.59 (d), 4.27(m)
8	Acetoin	1.38 (d)
9	Alanine	1.46 (d)
10	Lysine	1.43 (m), 1.50 (m), 1.72 (m), 1.89 (m), 1.92 (m)
11	Acetate	1.90 (s)
12	Butyrate	2.12 (t)
13	Glutamine	2.13 (m), 2.42 (m), 2.44 (m)
14	Acetoacetate	2.26 (s)
15	Pyruvate	2.35 (s)
16	Succinate	2.38 (s)
17	Citrate	2.52 (d), 2.66 (d)
18	Methionine	2.63 (t)
19	Trimethylamine	2.88 (s)
20	Creatine	3.03 (s), 3.92 (s)
21	Glucose	3.24 (dd), 3.48 (t), 3.54 (dd), 3.71 (t), 3.72 (dd), 3.83 (m), 3.90 (dd), 4.64 (d), 5.30 (d)
22	Glycerol	3.55 (dd), 3.64 (dd), 3.78 (m)
23	Glycine	3.54 (s)
24	Allantoin	5.38(s)
25	Fumararte	6.50 (s)
26	Tyrosine	6.88 (d), 7.18 (d)

27	Histidine	7.04 (s), 7.76 (s)
28	Phenylalanine	7.32 (d), 7.35 (t), 7.42 (t)
29	Formate	8.46(s)
30	U1	2.07 (s)
31	U2	8.44 (s)

s, singlet; d, double; t, triplet; q, quartet; m, multiple; dd, double of double.

U1/U2: unknown metabolite 1/2

Supplementary Table S2. Significantly altered metabolic pathways identified from pairwise comparisons between the three groups of mouse sera.

**Pathway**	**Group**		
	NCAC *vs.* NOR	CAC *vs.* NCAC	CAC *vs.* NOR
Citrate cycle (TCA cycle)	√	√	√
Butanoate metabolism	√		√
Alanine, aspartate and glutamate metabolism	√	√	√
Glyoxylate and dicarboxylate metabolism	√	√	√
Tyrosine metabolism	√	√	√
Glycine, serine and threonine metabolism	√	√	
Synthesis and degradation of ketone bodies	√		√
Glycerolipid metabolism	√		√
Glycolysis / Gluconeogenesis	√	√	√
Pyruvate metabolism	√	√	√
Phenylalanine, tyrosine and tryptophan biosynthesis			√
Phenylalanine metabolism			√
